# Differential Expression of Circulating Plasma miRNA-370 and miRNA-10a from Patients with Hereditary Hemorrhagic Telangiectasia

**DOI:** 10.3390/jcm9092855

**Published:** 2020-09-03

**Authors:** Lidia Ruiz-Llorente, Virginia Albiñana, Luisa M. Botella, Carmelo Bernabeu

**Affiliations:** 1 Centro de Investigaciones Biológicas Margarita Salas, Consejo Superior de Investigaciones Científicas (CSIC) and Centro de Investigación Biomédica en Red de Enfermedades Raras (CIBERER), 28040 Madrid, Spain; lidia.ruizl@uah.es (L.R.-L.); vir_albi_di@yahoo.es (V.A.); cibluisa@cib.csic.es (L.M.B.); 2 Department of Systems Biology, School of Medicine and Health Sciences, University of Alcalá, Alcalá de Henares, 28871 Madrid, Spain

**Keywords:** microRNA, biomarker, hereditary hemorrhagic telangiectasia (HHT), plasma, telangiectases, arteriovenous malformations (AVMs), angiogenesis, endoglin, activin receptor-like kinase 1 (ALK1), transforming growth factor beta (TGF-β), bone morphogenetic protein (BMP)

## Abstract

Hereditary hemorrhagic telangiectasia (HHT) is an autosomal dominant, vascular disorder that presents with telangiectases and arteriovenous malformations. HHT is a genetically heterogeneous disorder, involving mutations in endoglin (*ENG*; HHT1) and activin receptor-like kinase 1 (*ACVRL1*/*ALK1*; HHT2) genes that account for over 85% of all HHT patients. The current diagnosis of HHT patients remains at the clinical level, but many suspected patients do not have a clear HHT diagnosis or do not show pathogenic mutations in HHT genes. This situation has prompted the search for biomarkers to help in the early diagnosis of the disease. We have analyzed the plasma levels in HHT patients of selected micro-RNAs (miRNAs), small single-stranded RNAs that regulate gene expression at the transcriptional level by interacting with specific RNA targets. A total of 16 HHT1 and 17 HHT2 plasma samples from clinically confirmed patients and 16 controls were analyzed in this study. Total RNA was purified from plasma, and three selected miRNAs (miRNA-10a, miRNA-214, and miRNA-370), related to the pathobiology of cardiovascular diseases and potentially targeting *ENG* or *ALK1*, were measured by quantitative polymerase chain reaction. Compared with controls, levels of miRNA-370, whose putative target is *ENG*, were significantly downregulated in HHT1, but not in HHT2, whereas the levels of miRNA-10a, whose putative target is *ALK1*, were significantly upregulated in HHT2, but not in HHT1. In addition, the levels of miRNA-214, potentially targeting *ENG* and *ALK1,* did not change in either HHT1 or HHT2 patients versus control samples. While further studies are warranted, these results suggest that dysregulated plasma levels of miRNA-370 or miRNA-10a could help to identify undiagnosed HHT1 or HHT2 patients, respectively.

## 1. Introduction

Hereditary hemorrhagic telangiectasia (HHT) is an autosomal dominant, vascular disorder with a prevalence of approximately 1 in 8000 people worldwide [1,2]. HHT is characterized by the presence of telangiectases in skin and mucocutaneous tissue and arteriovenous malformations (AVMs), direct connections between arteries and veins without capillary beds, in internal organs [1]. Over 90% of all HHT patients present chronic epistaxis due to nasal telangiectases, whereas chronic bleeding from gastrointestinal telangiectases develops in at least 20% of patients. The presence of pulmonary, hepatic, and cerebral AVMs in HHT patients suggests that they are at risk of life-threatening hemorrhage and complications involving shunting, leading to stroke, high-output cardiac failure, or brain abscess [3,4,5].

HHT is genetically heterogeneous with heterozygous mutations in at least three known genes including endoglin (*ENG*) causing HHT1 [6], activin receptor-like kinase 1 (*ACVRL1* or *ALK1*), causing HHT2 [7], and mothers against decapentaplegic homolog 4 (*MADH4* or *SMAD4*) causing familial juvenile polyposis associated with HHT (JP/HT) [8]. In addition, mutations in the *GDF2* gene, encoding bone morphogenetic protein 9 (BMP9), were described as the cause of an HHT-like syndrome [9]. *ENG* and *ACVRL1* are the predominant genes whose mutations account for over 85% of all HHT patients [10,11] and are considered potential therapeutic targets [12]. The phenotypes generated by either *ENG* or *ACVRL1* mutations (HHT1 or HHT2, respectively) generally display distinct clinical manifestations, but an overlap of their clinical symptoms commonly occurs. Interestingly, all the genes mutated in HHT encode proteins that are involved in the signaling pathway of transforming growth factor beta (TGF-β)/bone morphogenetic protein (BMP). Thus, the auxiliary receptor endoglin associates with the signaling serine/threonine receptor ALK1 and both proteins are able to bind ligands like BMP9, leading to the phosphorylation and nuclear translocation of a Smad protein complex that includes Smad4 [12,13,14]. Because endothelial cells are functional targets of circulating BMP9, and predominantly express endoglin and ALK1, these are widely accepted as the target cells in HHT.

A deficient expression of the HHT genes has been postulated to underlie the molecular basis of HHT pathogenesis. Mono-allelic loss of expression leading to haploinsufficiency of the respective HHT proteins has been shown to dysregulate TGF-β/BMP signaling in endothelial cells, negatively impacting in cell proliferation, migration, and recruitment during vascular remodeling and angiogenesis [11,12]. In addition, in mice, homozygous knockdown of *ENG* or *ACVRL1* leads to HHT phenotypes [15,16,17]. More recently, using next-generation sequencing, Marchuk et al. [18] have been able to demonstrate the presence of low-frequency somatic mutations in telangiectases of HHT1 and HHT2 patients, suggesting that the bi-allelic loss of *ENG* or *ACVRL1* may also contribute to the development of vascular lesions.

MicroRNAs (miRNAs) are small noncoding RNAs (about 22 nucleotides in length) able to post-transcriptionally regulate gene expression by RNA interference [19,20]. They act as guides in complementary miRNA–mRNA complexes that recruit enzymatic species to silence mRNAs by mRNA cleavage (perfect complementarity) or ribosome destabilization (nonperfect complementarity). In this process, Drosha is a ribonuclease that plays a key role for miRNA biogenesis and regulates the TGF-β/BMP pathway by interacting with the Smad protein family which, in turn, modulates gene expression via miRNAs [21]. Interestingly, mice lacking Drosha in the vascular endothelium develop a vascular phenotype resembling HHT including dilated and disorganized vasculature, AVMs, and hemorrhages [22]. In this regard, the emerging role of miRNAs in human diseases [23,24] has prompted a few studies searching for specific miRNAs in HHT patients [25,26,27]. However, the expression of miRNAs associated with HHT remains mostly unexplored. Our aim in this work was to identify dysregulated miRNAs in plasma samples from HHT patients through quantitative polymerase chain reaction compared with controls. We find i) a downregulation in HHT1, but not in HHT2, patients of miRNA-370, which is predicted to target *ENG*; and ii) an upregulation in HHT2, but not in HHT1, patients of miRNA-10a, whose putative target is *ALK1*. These results suggest that dysregulated plasma levels of miRNA-370 or miRNA-10a are potential biomarkers that could help to identify undiagnosed HHT1 or HHT2 patients, respectively. In addition, they also may contribute to a better understanding of the complex biological processes associated with the development of HHT.

## 2. Material and Methods

### 2.1. Algorithms for miRNA-target Predictions

Computational prediction of miRNA targets was carried out using three target prediction programs which use different algorithms: (i) microRNA (www.microRNA.org) [28]; (ii) Target Scan (http://www.targetscan.org/vert_72/) [29]; and (iii) MicroCosm (https://tools4mirs.org/software/mirna_databases/microcosm-targets/) [30]. The mirSVR score was used as a regression method for predicting likelihood of target mRNA downregulation from sequence and structure features in microRNA/mRNA predicted target sites.

### 2.2. Patients

A total of 34 HHT patient plasma samples were evaluated by quantitative reverse transcriptase–polymerase chain reaction (qRT-PCR). Seventeen of them were HHT1, and the remaining seventeen patients were HHT2. Plasma samples from 16 control healthy subjects were also assayed to establish the normal range of microRNAs (miRNAs). In all three groups, donors from different sex and age were included. Peripheral venous blood samples were collected with ethylenediamine tetra-acetic acid (EDTA) as anticoagulant. Blood samples were centrifuged at 15,000 *g* for 15 min, and the resulting plasma was stored at −80 °C until analysis. Written informed consent was obtained from all the participants, or their legally authorized representative, in this study, and the protocol was supervised and received full approval from our Institutional Review Board (IRB) of the Spanish National Research Council (CSIC) with the ethical code number 075/2017. All HHT patients included in the present study were clinically diagnosed following the Curaçao criteria [3], and their genetic mutations were identified by sequencing. Their genetic characteristics and clinical manifestations are summarized in Table 1.

### 2.3. Extraction of miRNAs from Plasma Samples

miRNAs were extracted with the miRNeasy Micro kit (Qiagen, Hilden, Germany; #217084) according to the manufacturer’s instructions and following a previously described protocol [25,31]. Briefly, 50 µL plasma samples from HHT patients were homogenized with 1 mL QIAzol^®^ Lysis Reagent and 6.25 × 10^−3^ fmol/µL of the spike-in control cel-miR-39-3p (5′ UCA CCG GGU GUA AAU CAG CUU G 3′). Next, the homogenate was incubated at room temperature for 5 min, and then 200 µL chloroform was added to each sample, followed by centrifugation at 12,000× *g* for 5 min at 4 °C. Equal volumes from the upper aqueous phase were transferred to new tubes and mixed by pipetting with 1.5 volumes of 100% ethanol; finally total miRNAs were eluted in 40 µL RNase-free water.

### 2.4. Quantitative Reverse Transcriptase–Polymerase Chain Reaction (qRT-PCR) Analysis of miRNAs

qRT-PCR was performed to validate selected miRNAs. They were reverse-transcribed into cDNA using TaqMan™ MicroRNA Reverse Transcription kit (Thermo Fisher Scientific, #4366596) following the manufacturer´s protocol. The reaction components were mixed with the corresponding reverse transcriptase (RT) primers of hsa-miR-370-3p, hsa-miR-214-3p, hsa-miR-10a-3p, hsa-miR-16-5p, and cel-miR-39-3p (Thermo Fisher Scientific, Waltham, MA, USA; #4427975; Assay ID 002275, 002306, 002288, 000391, and 000200, respectively). RT reaction was carried out as follows: 16 °C for 30 min, 42 °C for 30 min, 85 °C for 5 min, and 4 °C hold. The resulting cDNAs were used for quantitative real-time PCR experiments using Taqman Universal PCR Master Mix, no AmpErase^®^ UNG (Thermo Fisher Scientific, Waltham, MA, USA; #4324018), and the specific PCR primers (references detailed above). Thermal cycling was performed on LightCycler^®^ 96 detection system (Roche) as follows: 95 °C for 10 min, 40 cycles of 95 °C for 15s and 60 °C for 1 min. Relative quantification of individual miRNA expression was carried out with the 2-ΔΔCT method and normalized against two internal controls, miR-16-5p and the spike-in miRNA cel-miR-39-3p. In studies focused on cardiovascular disease, miR-16-5p is one of the miRNAs with the best performance as normalizer [32], whereas cel-miR-39-3p is one of the most used and reliable spike-in controls [25].

### 2.5. Statistical Analysis

HHT patient and control groups were compared using Kruskal–Wallis test. Subsequent Mann–Whitney U tests were run to test for pairwise comparisons in a post hoc fashion; significance values were adjusted by the Bonferroni correction for multiple tests. Statistical analyses were carried out with the IBM SPSS Statistics version 25 (Windows10 64-bit) software (IBM Corp., Armonk, NY, USA). Box Whisker plots show median (central line), upper and lower quartiles (box), and range excluding outliers (whiskers). Asterisks indicate statistically significant values between selected conditions (* *p* < 0.05; ** *p* < 0.01; ns, not significant).

## 3. Results

### 3.1. Identification of miRNA-10a, miRNA-214, and miRNA-370, Potentially Related to HHT, Using in Silico and Literature Data

Recently, studies on circulating miRNAs as potential biomarkers in different types of diseases [33], including HHT [21,27], have received increasing attention. In order to identify specific biomarkers of HHT, we searched for miRNAs involved in angiogenesis and vascular homeostasis and predicted to target *ENG* and/or *ALK1* using three robust target prediction programs which use different algorithms: (i) microRNA [28]; (ii) Target Scan [29]; and (iii) MicroCosm [30]. Using this stringent approach, we selected miRNA-370, miRNA-10a, and miRNA-214 for further analyses.

miRNA-370 was considered to be very relevant to endoglin function since it was the only miRNA predicted to target *ENG* with a good mirSVR score (−0.1740), according to the microRNA database (www.microRNA.org). This website also describes miRNA-370 as an evolutionary well-conserved miRNA because its alignment with *ENG* is found in other species as well. Likewise, algorithms from Target Scan and MicroCosm programs indicate that *ENG* is predicted to be targeted by miRNA-370. In fact, the negative regulation of *ENG* by miRNA-370 has been validated in ovarian cancer cells where miRNA-370 suppresses their proliferation and promotes chemosensitivity to cisplatin by negatively regulating *ENG* [34]. Based on predictions of microRNA webs, miRNA-370 not only regulates *ENG*, but also other gene products with essential roles in endothelial cell biology and angiogenesis, some of which have been already validated as targets in the literature. One of these proteins is TGF-β receptor type 2 (TGFBR2) that is negatively regulated by miRNA-370. By acting via TβRII, miRNA-370 plays a potential role in hepatic ischaemia-reperfusion injury and indeed, its inhibition efficiently attenuates liver damage [35]. In addition, upregulation of miRNA-370 might promote the repair of amputated fingers by regulating angiogenesis through targeting Forkhead box protein O1 (FOXO1) [36]. miRNA-370 can also induce growth and tube formation inhibition, and apoptosis in endothelial cells [37,38]. The miRNA-370-induced endothelial effects may explain its anti-angiogenic activity, as well as its developmental regulation of cerebral aneurysms [38,39,40]. Noteworthy, the effects miRNA-370 are mediated by targeting, at least, a receptor for vascular endothelial growth factor (VEGF), which is the major driver of angiogenesis [38,39].

We next focused our interest on miRNA-10a and miRNA-214 because both are predicted to target *ENG* or *ALK1*, and their dysregulated expression in HHT patients with pulmonary AVMs (pAVMs) has been reported [26] Thus, *ENG* has been revealed, by MicroCosm and Target Scan, as a confident target of miRNA-214. A search in microRNA.org revealed that miRNA-214 is also expected to bind ALK1 mRNA at two different sites with high mirSVR scores (−0.1277 and −0.5112), and ranks in the first position among all miRNAs potentially targeting ALK1. Moreover, by targeting matrix metalloproteinase 8 (MMP8), hepatoma-derived growth factor (HDGF), brain-specific angiogenesis inhibitors (BAIs), and other vascular-related genes, miRNA-214 contributes to the pathogenesis of various cardiovascular conditions, including ischaemic heart diseases, angiogenesis, and cardiac hypertrophy [41,42,43]. Of note, miRNA-214 is a response element to hypoxia in patients with pulmonary arterial hypertension (PHA), a complication of HHT. Moreover, inhibition of miRNA-214 can ameliorate the symptoms of PHA in animal models, suggesting its use for the prevention and treatment of PHA in humans [41].

Bioinformatics analysis of microRNA database showed that miRNA-10a does not target *ENG*, but appears to regulate *ALK1* with a good mirSVR score (−0.3837). In addition, miRNA-10a targets endothelial gene products such as VEGF receptor 1 (FLT1), β-catenin, GATA-binding factor 6 (GATA6), or mib-1, which may account for its active regulatory role in endothelial cell biology and angiogenesis [44,45,46].

### 3.2. Circulating Levels of miRNA-370, miRNA-10a, and miRNA-214 in HHT1 and HHT2 Patient Plasma

Taken together, the above studies suggest that miRNA-370, miRNA-10a, and miRNA-214 are predicted to target not only *ENG* and/or *ALK1*, but also other relevant gene products involved in vascular functions related to the pathophysiology of HHT. Therefore, the chosen miRNAs were next validated by qRT-PCR in the plasma samples from a cohort of HHT1 and HHT2 patients and healthy controls in order to assess their possible diagnostic or biomarker value.

#### 3.2.1. Circulating Levels of miRNA-370 are Decreased in HHT1 Patient Plasma

**Figure 1:**
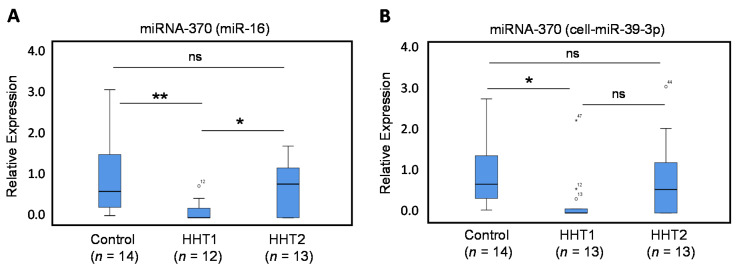
Quantitative Reverse Transcriptase–Polymerase Chain Reaction (qRT-PCR) of miRNA-370. Total plasma RNA was isolated from HHT1 and HHT2 patients and control subjects. Relative expression levels of miR-370 were measured by qRT-PCR using miR-16 (**A**) or cel-miR-39-3p (**B**) as normalizers. The number of samples analyzed is indicated in parentheses. Symbols outside the box plot represent extreme values (°) and outliers (*) with their corresponding sample numbers. Experiments were performed in triplicates. (* *p* < 0.05; ** *p* < 0.01; ns, not significant).

The expression levels of miRNA-370, as measured by qRT-PCR, were found to be significantly lower in plasma samples from HHT1 patients (Figure 1), using miRNA-16 (Figure 1A; *p* = 0.001) or cel-miR-39-3p (Figure 1B; *p* = 0.004), as normalizers when compared with healthy controls. The levels of miRNA-370 in HHT1 patients were also significantly lower than those in HHT2 patients when using miR-16 as a normalizer (Figure 1A; *p* = 0.019), and showed a not significant but clear decreasing trend versus HHT2 samples when using cel-miR-39-3p as a normalizer (Figure 1B; *p* = 0.116). By contrast, the expression levels of miRNA-370 in HHT2 patients were not significantly affected compared with healthy control individuals. The specific downregulation in HHT1 patients suggests that the decreased levels of miRNA-370 may have potential diagnostic utility as an HHT1 biomarker. Interestingly, since miRNA-370 is predicted to target *ENG* to downregulate its expression [34] and a deficient *ENG* expression underlies the pathogenicity of HHT1, both results also suggest the existence of a common link between the expression levels of miRNA-370 and endoglin in HHT1 patients.

#### 3.2.2. Circulating Levels of miRNA-10a, but not of miRNA-214, are Dysregulated in HHT2 Patient Plasma

**Figure 2:**
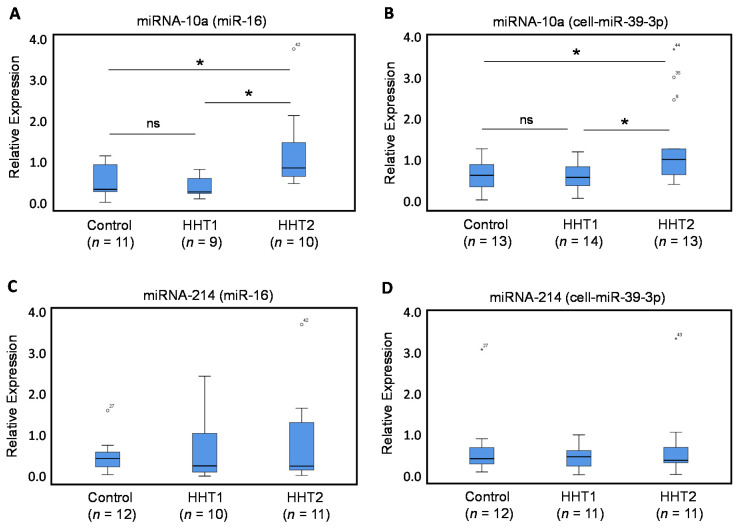
qRT-PCR of miRNA-10a and miRNA-214. Total plasma RNA was isolated from HHT1 and HHT2 patients and control subjects. Relative expression levels of miRNA-10a (**A**,**B**) and miRNA-214 (**C**,**D**), measured by qRT-PCR using miR-16 (**A**,**C**) or cel-miR-39-3p (**B**,**D**) as normalizers. Symbols outside the box plot represent extreme values (°) and outliers (*) with their corresponding sample numbers. The number of samples analyzed is indicated in parentheses. Experiments were performed in triplicates. (* *p* < 0.05; ns, not significant).

As measured by qRT-PCR, the expression levels of miRNA-10a were significantly higher in plasma samples from HHT2 patients (Figure 2), using miRNA-16 (Figure 2A; *p* = 0.026) or cel-miR-39-3p (Figure 2B; *p* = 0.024), as normalizers, when compared with healthy controls. By contrast, the expression levels of miRNA-10a in HHT1 patients were not significantly affected compared with healthy control individuals. Even more, the levels of miRNA-10a in HHT2 patients were significantly higher than those in HHT1 patients when using miR-16 (Figure 2A; *p* = 0.03) or cel-miR-39-3p (Figure 2B; *p* = 0.018), as normalizers. The specific upregulation in HHT2 patients suggests that the increased levels of miRNA-10a may have potential diagnostic utility as an HHT2 biomarker. Interestingly, since miRNA-10 potentially targets ALK1 and a deficient ALK1 expression underlies the pathogenicity of HHT2, these results also suggest the existence of a common link between the expression levels of both miRNA-10a and ALK1 in HHT2 patients. Plasma levels of miRNA-214 were also measured by qRT-PCR, using miR-16 or cel-miR-39-3p as normalizers (Figure 2C,D). However, no statistically significant differences were found in the expression levels of miRNA-214 from either HHT1 or HHT2 patients, when compared with healthy controls, or between each other (Figure 2C,D).

## 4. Discussion

Over 1900 miRNAs have been identified in humans and many of them have been reported to be involved in a variety of human diseases, biological functions, and signaling pathways, including the TGF-β/BMP route [23,24]. Among these, there are a growing number of miRNAs with high potential to be used as biomarkers in plasma and/or serum to clinically diagnose or provide accurate prognosis for survival in patients with cardiovascular diseases [47]. HHT is an autosomal dominant, genetic disorder in which patients develop hemorrhagic vascular lesions called telangiectases and AVMs. A few reports have previously searched for specific miRNAs in plasma [25,26] or peripheral mononuclear cells [27] from HHT patients. Recently, it has been reported that Drosha, a key enzyme in miRNA biogenesis, regulates vascular development and homeostasis via the TGFβ/BMP pathway, and rare missense mutations in the Drosha gene may predispose carriers to HHT [21,22]. These findings support the hypothesis that miRNAs play a functional role in HHT. However, the expression of miRNAs and their potential application as biomarkers in HHT remains mostly unexplored. In addition, how miRNAs might be involved in HHT development is not fully understood. Our goal was to measure several miRNAs in HHT patient-derived plasma as in previous studies [25,26], but with the novel strategy of selecting miRNAs potentially targeting the predominant HHT genes. We report here for the first time that plasma miRNA-370 levels are downregulated in HHT1, but not in HHT2, patients; while plasma miRNA-10a levels are increased in HHT2, but not in HHT1, patients. While further studies in a large cohort of patients are warranted, this finding suggests that miRNA-370 and miRNA-10a could be used as biomarkers with potential application in HHT diagnosis [48]. Actually, this possibility would be of great interest because there is a need to diagnose HHT in those individuals who do not present with all of the typical symptoms, such as in asymptomatic children and young adults [4,5]. When the HHT gene mutation of the family is known, the genetic testing is the choice option in children and teenagers; the genetic results being definite for either the positive diagnosis or exclusion [10,49]. However, in the absence of a known family mutation, genetic determinations imply the sequencing of at least *ENG* and *ACVRL1* genes, a process that is time-consuming and expensive. Even so, in 10–15% of the subjects, the mutation is not identified. Therefore, it would be useful to have alternative tools to ideally allow an earlier, faster, cheaper, and easier HHT diagnosis. In this sense, several potential biomarkers, including miRNAs, have been described in HHT [48]. Thus, the specific downregulation of miRNA-370 in HHT1 and upregulation of miRNA-10a in HHT2 can be added to the reported dysregulated expression of miRNA-27a, miRNA-205 [25], miRNA-210 [26], miRNA-28-5p, and miRNA-361-3p [27] in different HHT subsets, as shown in Table 2. Unfortunately, due to the different experimental approach and material source (plasma or peripheral mononuclear cells (PMNCs)) used in each of these studies, no comparative conclusions could be drawn. Regarding miRNA-10a and miRNA-214, a previous report has suggested their dysregulated expression using miRNA array analysis in a pool of genotyped and nongenotyped HHT patients with pAVMs [26]. However, to the best of our knowledge, no conclusive, quantitative measurements of these miRNAs have been made so far. In this work, we find that miR10a expression is specifically upregulated in HHT2, whereas the levels of miRNA-214 were unaffected in either HHT1 or HHT2 patients. Nonetheless, a direct comparison between the results by Zhang et al. (2013) and this work is not feasible since the HHT population analyzed in each case is different and pAVMs are present in up to 50% of the HHT population with a predominant association with HHT1 patients [5,50]. Future investigations should be addressed to unify the source of the patient samples and its methodological processing in order to identify miRNAs that could serve as reliable biomarkers in HHT.

A good biomarker is a defined characteristic that is measured as an indicator of normal biological processes, pathogenic processes, or responses to an exposure or intervention [51,52]. This broad definition encompasses molecular characteristics such as the dysregulated expression of miRNA-370 and miRNA-10a presented in this work. Once a potential biomarker is identified, the next step is to assure that it can be measured reliably and precisely [51,52]. In this regard, further validation studies using a larger cohort of HHT patients are needed, including disease stratification analysis to assess the possible correlation between the levels of miRNA-370 and miRNA-10a and the severity of symptoms. In addition, it would be interesting to measure the circulating subsets of miRNA-370 and miRNA-10a such as those in plasma exosomes, plasma free miRNAs, or mononuclear cells from HHT patients.

**Figure 3:**
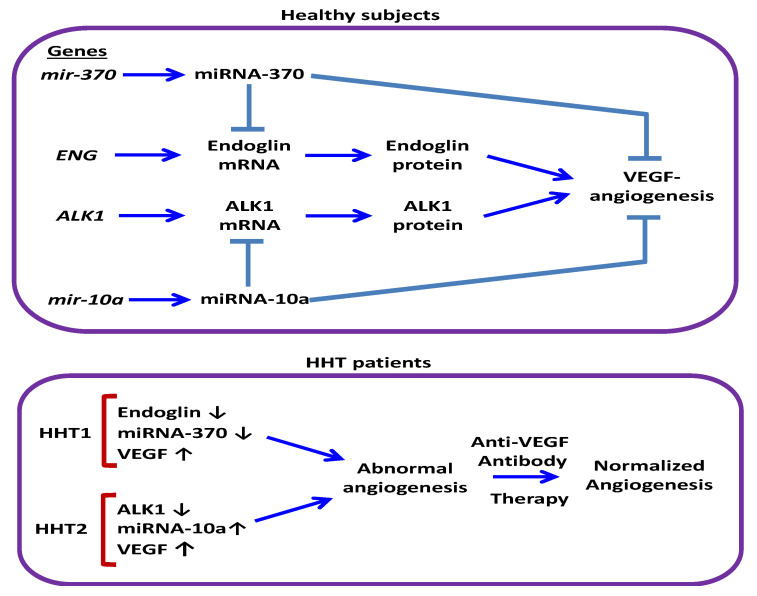
Hypothetical model for the involvement of miRNA-370 and miRNA-10a in HHT. Top, in healthy subjects, basal levels of miRNA-370 and miRNA-10a are predicted to target endoglin, ALK1, as well as the VEGF/VEGFR pathway, thus regulating angiogenesis. Bottom, in HHT patients, heterozygous mutations lead to a deficient expression of endoglin (HHT1) or ALK1 (HHT2), associated with increased levels of VEGF and the dysregulated expression of miRNA-370 and miRNA-10a, resulting in abnormal angiogenesis. Treatment with antibodies to VEGF (bevacizumab) contributes to angiogenesis normalization. ALK1: activin receptor-like kinase 1; VEGF: vascular endothelial growth factor; VEGFR: VEGF receptors.

To understand the value of a biomarker, it is necessary to know the pathophysiological relationship between the biomarker and the relevant clinical endpoint [51]. That miRNAs have a functional impact on HHT development is supported by recent next-generation sequencing studies of DROSHA, a key ribonuclease involved in miRNA biogenesis [21]. Thus, four rare variants of DROSHA, predicted to damage protein function (P32L, P100L, K226E, R279L), are present at a much higher frequency in HHT patients compared with healthy controls, suggesting that they may contribute to the HHT onset [22]. However, the function of miRNAs associated with HHT, such as miRNA-370 and miRNA-10a, remains to be elucidated. Both miRNA-370 and miRNA-10a regulate angiogenesis [36,38,53], a VEGF-dependent biological process involved in the pathophysiology and therapy of HHT [54,55], by targeting, at least, the VEGF receptors (VEGFR) KDR (kinase insert domain receptor) [38,39] and FLT1 (fms-related tyrosine kinase 1) [45], respectively. In addition, circulating VEGF levels are increased in HHT1 and HHT2 patients compared with the control population [48,56,57], and therapeutic antibodies to VEGF (bevacizumab) are currently used to alleviate HHT symptoms [55,58]. Interestingly, the VEGF/VEGFR pathway can be activated by endoglin or ALK1, thus promoting angiogenesis [59,60]. Therefore, by regulating VEGF-dependent angiogenesis, miRNA-370 and miRNA-10a may potentially impact the pathophysiology of HHT1 and HHT2. Of note, several experimental reports have already demonstrated the involvement of miRNA-370 in different functions of endothelial cells, the target cells in HHT where endoglin is predominantly expressed [38,40]. Thus, miRNA-370 suppresses retinal capillary endothelial cell growth and apoptosis [38], inhibits the angiogenic activity of endothelial cells, and reduces microvessel density and sprouting in vivo, whereas a miRNA-370 inhibitor promotes endothelial sprout formation [40]. The antiangiogenic activity of miRNA-370 is fully compatible with the key functional role of its putative target endoglin in endothelial cells, as endoglin promotes angiogenesis in vivo and in vitro, at least, by stimulating proliferation and anti-apoptotic activity of endothelial cells [61]. Noteworthy, miRNA-370 and miRNA-10a are predicted to target *ENG* and *ALK1*, respectively, thus inhibiting its expression. While targeting of *ENG* by miRNA-370 in nonendothelial cells has been previously validated [34], to the best of our knowledge, the validation of *ALK1* targeting by miRNA-10a has not been reported. One limitation of our study is that the predicted targeting of *ENG* and *ALK1* by miRNA-370 and miRNA-10a, respectively, has not been experimentally validated in human endothelial cells. Whether the altered levels of circulating miRNA-370 or miRNA-10a in HHT1 or HHT2 patients contribute to the regulated expression and function of endoglin or ALK1 in the endothelium remains an interesting avenue of research in the future. Because the levels of miRNA-370 or miRNA-10a are specifically dysregulated in patients who present a deficient expression of *ENG* (HHT1) or *ALK1* (HHT2), respectively, a possible link between the expression levels of the HHT genes and those miRNAs can be postulated (Figure 3). Since haploinsufficiency underlies the pathogenic mechanism in HHT1 and HHT2, it can be speculated that the dysregulated expression of miRNA-370 and miRNA-10a may affect HHT development by modulating the expression levels of *ENG* or *ALK1*. However, there is an opposite effect in the expression levels of miRNA-370 (upregulated) versus miRNA-10a (downregulated), suggesting a contrary effect on the expression levels of *ENG* or *ALK1*. Moreover, the expression levels of miRNA-214, which is predicted to target *ENG* and *ALK1*, are not significantly affected in our HHT population study. These apparent discrepancies are likely explained by the fact that the genes encoding miRNA-370 (MIR370; chromosome 14), miRNA-10a (MIR10A; chromosome 17), and miRNA-214 (MIR214; chromosome 1) are differentially regulated by distinct gene expression programs. Furthermore, miRNA-370 and miRNA-10a may target not only *ENG* or *ALK1*, but also the other genes, especially those relevant to vascular development, which may turn out be of greater relevance in the complex mechanism of lesion development in HHT.

## 5. Conclusions

In summary, here we find that decreased levels of miRNA-370 or increased levels of miRNA-10a in plasma from HHT patients represent novel biomarkers that could help to identify undiagnosed patients within the HHT1 or HHT2 subsets, respectively. Both miRNA-370 and miRNA-10a can regulate angiogenesis, a VEGF-dependent biological process involved in the pathophysiology and therapy of HHT, by targeting, at least, VEGF receptors. Additionally, miRNA-370 and miRNA-10a are predicted to target *ENG* and *ALK1* genes, respectively, whose deleterious mutations lead to HHT1 or HHT2, respectively. These findings open up a new research avenue to better understand the functional impact of dysregulated miRNAs in HHT development. Future independent studies remain to be performed in order to further validate the role of miRNA-370 and miRNA-10a as biomarkers in HHT, as well as to investigate the functional and pathophysiological significance of these newly discovered miRNA-370/HHT1 and miRNA-10a/ALK1 associations.

## Figures and Tables

**Table 1:** Summary of genotypes and mutations of hereditary hemorrhagic telangiectasia (HHT) of patients ^1^.

HHT Type	Patient#	Genotype	Mutation
HHT1	P#1.1	*ENG*	5′ UTR (gene promoter) c.-127 G>A
HHT1	P#1.2	*ENG*	5′ UTR (gene promoter) c.-127 G>A
HHT1	P#1.3	*ENG*	5′ UTR (gene promoter) c.-58 G>A
HHT1	P#1.4	*ENG*	5′ UTR (gene promoter) c.-58 G>A
HHT1	P#1.5	*ENG*	5′ UTR (gene promoter) c.-58 G>C
HHT1	P#1.6	*ENG*	Intron 1 c.68-2A>T
HHT1	P#1.7	*ENG*	Intron 1 c.68-2A>T
HHT1	P#1.8	*ENG*	Intron 1 c.68-2A>T
HHT1	P#1.9	*ENG*	Exon 4 c.392 C>T; p.Pro131Leu
HHT1	P#1.10	*ENG*	Exon 5 c.588 G>A; p.Trp196 *
HHT1	P#1.11	*ENG*	Exon 5 c.588 G>A; p.Trp196 *
HHT1	P#1.12	*ENG*	Exon 5 c.617delG; p.G206AfsX16
HHT1	P#1.13	*ENG*	Exon 5 c.617delG; p.G206AfsX16
HHT1	P#1.14	*ENG*	Exon 7 c.967_968del GT; p.V323fs *
HHT1	P#1.15	*ENG*	Exon 7 c.967_968delGT; p.V323fs *
HHT1	P#1.16	*ENG*	Exon 11 c.1434_1435 del AG p. R478fs *
HHT2	P#2.1	*ALK1*	Exon 6 c.673_674delAG; p.S225fs
HHT2	P#2.2	*ALK1*	Exon 6 c.673_674delAG; p.S225fs
HHT2	P#2.3	*ALK1*	Exon 6 c.635 G>A; p.R212H
HHT2	P#2.4	*ALK1*	Exon 7 c.889delC; H297fs *
HHT2	P#2.5	*ALK1*	Exon 7 c.921-927dupATGCGGC; p.L310fs
HHT2	P#2.6	*ALK1*	Exon 7 c. 926 G>A; p.G309A
HHT2	P#2.7	*ALK1*	Exon 7 c.941 A>C; p.His314Pro
HHT2	P#2.8	*ALK1*	Exon 7 c.988 G>T; p.D330Y
HHT2	P#2.9	*ALK1*	Exon 7 c.1027 C>T; p.Q374X
HHT2	P#2.10	*ALK1*	Exon 7 c.1027 C>T; p.Q374X
HHT2	P#2.11	*ALK1*	Exon 7 c.1027 C>T; p.Q374X
HHT2	P#2.12	*ALK1*	Exon 7 c.1030 C>T; p.C344R
HHT2	P#2.13	*ALK1*	Exon 8 c.1120 C>T; p.R374W
HHT2	P#2.14	*ALK1*	Exon 8 c.1120 C>T; p.Arg374Trp
HHT2	P#2.15	*ALK1*	Exon 8 c.1232 G>A; p.Arg411Gln
HHT2	P#2.16	*ALK1*	Exon 10 c.1435 C>T; p.Arg479X

^1^ A total of 33 HHT patients were included in this microRNA analyses. All HHT patients were clinically diagnosed following the Curaçao criteria [3]. Sixteen patients were genetically diagnosed as HHT1 as they harbor a mutation in *ENG*, whereas the remaining 17 patients were HHT2 with pathogenic mutations in *ALK1*; fs—frameshift mutation; Asterisks (*) represent stop codons.

**Table 2:** miRNAs associated with HHT ^1^.

miRNA	HHT1	HHT2	HHT (Pool)	Blood Sample	Reference
miRNA-370	↓	↔	ND	Plasma	Present work
miRNA-10a	↔	↑	ND	Plasma	Present work
miRNA-28-5p	ND	ND	↓	PMNCs	Cannavicci et al., 2019
miRNA-361-3p	ND	ND	↓	PMNCs	Cannavicci et al., 2019
miRNA-210	ND	ND	↑	Plasma	Zhang et al., 2013
miRNA-210	ND	ND	(with PAVMs)	Plasma	Zhang et al., 2013
miRNA-27a	↑	↑	↔	Plasma	Tabruyn et al., 2013
miRNA-205	↓	↓	(without PAVMs)	Plasma	Tabruyn et al., 2013

^1^ Total RNA from either plasma or peripheral mononuclear cells (PMNCs) was isolated, and levels of specific miRNAs were quantified, as indicated in the corresponding references. Increased (↑), decreased (↓), or unaffected (↔) levels of miRNAs are shown. HHT, hereditary hemorrhagic telangiectasia. ND, not determined.
